# Supplementary material for: Mobility and its sensitivity to fitness differences determine consumer–resource distributions
Source: R Soc Open Sci. 2020 Jun 17;7(6):200247. doi: 10.1098/rsos.200247 (PMC7353973; doi:10.1098/rsos.200247)
Supplement: A proof of the uniqueness and stability of the equilibriums in the absence of consumer demography; The relationships among mobility, fitness sensitivity and the time for the system to reach equilibrium in the absence of consumer demography; The proof of the relationship between the fitness sensitivi [file rsos200247supp1.pdf]

Mobility and its sensitivity to fitness differences determine consumer-resource distributions

Jing Jiao, Louise Riotte-Lambert, Sergei Pilyugin, Michael Gil and Craig Osenberg

Appendix 1: A proof of the uniqueness and stability of the equilibriums in the absence of consumer demography

The model in the absence of consumer demography is:

$$\dot{R_H} = r_H R_H \left(1 - \frac{R_H}{K_H}\right) - \alpha R_H C_H \quad \text{(S1)}$$

$$\dot{R_L} = r_L R_L \left(1 - \frac{R_L}{K_L}\right) - \alpha R_L C_L \quad \text{(S2)}$$

$$\dot{C_H} = C_L \beta e^{\lambda(w_H - w_L)} - C_H \beta e^{\lambda(w_L - w_H)} \quad \text{(S3)}$$

$$C_L = 2C_T - C_H \quad \text{(S4)}$$

in which $\lambda = \gamma\beta, w_H = c\alpha R_H - \mu,\ w_L = c\alpha R_L - \mu$. For simplicity but without influencing the results qualitatively, here we assume $\gamma = 1$.

The Jacobian matrix at positive equilibrium (obtained by setting all Eq.s S1-3 equal to 0) is:

$$\begin{bmatrix} -\frac{r_H R_H^*}{K_H} & 0 & -\alpha R_H^* \\ 0 & -\frac{r_L R_L^*}{K_L} & \alpha R_L^* \\ 2\lambda^2 c\alpha C_H^* e^{-\lambda c\alpha\Delta} & -2\lambda^2 c\alpha C_H^* e^{-\lambda c\alpha\Delta} & -\frac{2\lambda C_T}{C_L^*} e^{-\lambda c\alpha\Delta} \end{bmatrix},$$

where $\Delta = R_H^* - R_L^*$ and $C_T = (C_H + C_L)/2$. From the above matrix, we can get the third-order polynomial of eigenvalue z:

$$z^3 + \left(\frac{2\lambda C_T}{C_L^*} e^{-\lambda c\alpha\Delta} + \frac{r_H R_H^*}{K_H} + \frac{r_L R_L^*}{K_L}\right) z^2 + \varphi z^1 + 2\lambda^2 c\alpha C_H^* e^{-\lambda c\alpha\Delta}\left(\frac{r_H R_H^*}{K_H}\alpha R_L^* + \frac{r_L R_L^*}{K_L}\alpha R_H^*\right) z^0$$

where $\varphi = \frac{r_H r_L R_H^* R_L^*}{K_H K_L} + \frac{r_H R_H^*}{K_H}\frac{2\lambda C_T}{C_L^*} e^{-\lambda c\alpha\Delta} + \frac{r_L R_L^*}{K_L}\frac{2\lambda C_T}{C_L^*} e^{-\lambda c\alpha\Delta} + 2\lambda^2 c\alpha^2 R_L^* C_H^* e^{-\lambda c\alpha\Delta} +$
$2\lambda^2 c\alpha^2 R_H^* C_H^* e^{-\lambda c\alpha\Delta})$

Using the Routh-Hurwitz criterion, and due to the fact that all the above coefficients of z^0, z^1, z^2 and $z^3 > 0$, any positive equilibrium (Eq. S1-4) is stable.

In what follows, we prove the uniqueness of the positive solution. By setting Eq. S1 and S2 = 0, we get: R_H^* = max (K_H ($1 - \frac{\alpha C_H^*}{r_H}$), 0), R_L^* = max (K_L ($1 - \frac{\alpha C_L^*}{r_L}$), 0), so R_H^* decreases in C_H^* and

R_L^* decreases in C_L^*.

To ensure that the solution is positive, we must have $R_H^*>0$ and $R_L^*>0$, which is equivalent to: K_H ($1 - \frac{\alpha C_H^*}{r_H}$) > 0 and K_L ($1 - \frac{\alpha C_L^*}{r_L}$) > 0. Rearranging these two inequalities yields:

$$C_H^* < \frac{r_H}{\alpha} \text{ and } C_L^* < \frac{r_L}{\alpha} \qquad \text{(S5)}$$

We then let Eq. S3 = 0, that is, $C_L\beta e^{\lambda(w_H - w_L)} - C_H\beta e^{\lambda(w_L - w_H)} = 0$. After rearrangement, it becomes:

$$0 = (2C_T - C_H^*)\beta e^{\lambda(w_H - w_L)} - C_H^*\beta e^{\lambda(w_L - w_H)} = G(C_H^*) \qquad \text{(S6)}$$

where G represents a function of C_H^*. Because G decreases in C_H^*, so there exists at most one value C_H^* to make $G(C_H^*) = 0$.

Replacing C_L^* by $2C_T - C_H^*$ in S5 and rearranging the inequality, we get:

$$2C_T - \frac{r_L}{\alpha} < C_H^* < \frac{r_H}{\alpha} \tag{S7}$$

To ensure positivity of R_H^* and R_L^*, C_T must satisfy:

$$\frac{r_L}{\alpha} \leq 2C_T < \frac{r_L}{\alpha} + \frac{r_H}{\alpha} \tag{S8}$$

Based on (S7) and the uniqueness of a positive C_H^* in (S6), we must have $G(2C_T - \frac{r_L}{\alpha}) > 0$ and $G(\frac{r_H}{\alpha}) < 0$.

By replacing $C_L^* = 2C_T - C_H^* = \frac{r_L}{\alpha}$ when $C_H^* = 2C_T - \frac{r_L}{\alpha}$, $R_H^* = K_H\left(1 - \frac{\alpha C_H^*}{r_H}\right) = K_H\left(1 - \frac{\alpha(2C_T - \frac{r_L}{\alpha})}{r_H}\right)$, $R_L^* = K_L\left(1 - \frac{\alpha C_L^*}{r_L}\right) = K_L(1-1) = 0$, we can get:

$G\,(2C_T - \frac{r_L}{\alpha}) = \frac{r_L}{\alpha}\beta e^{\lambda\left(c\alpha K_H\left(1 - \frac{2C_T\alpha - r_L}{r_H}\right)\right)} - \left(2C_T - \frac{r_L}{\alpha}\right)\beta e^{-\lambda\left(c\alpha K_H\left(1 - \frac{2C_T\alpha - r_L}{r_H}\right)\right)} > 0$. Rearranging the above inequality, we get one necessary condition for $G\,(2C_T - \frac{r_L}{\alpha}) > 0$:

$$\frac{2\alpha C_T - r_L}{r_L} < e^{2\lambda c\alpha K_H(1 - \frac{2C_T\alpha - r_L}{r_H})} \tag{S9}$$

By replacing $C_L^* = 2C_T - \frac{r_H}{\alpha}$ when $C_H^* = \frac{r_H}{\alpha}$, $R_H^* = K_H\left(1 - \frac{\alpha C_H^*}{r_H}\right) = K_H(1-1) = 0$, $R_L^* = K_L\left(1 - \frac{\alpha C_L^*}{r_L}\right) = K_L\left(1 - \frac{\alpha(2C_T - \frac{r_H}{\alpha})}{r_L}\right)$, we get: $G(\frac{r_H}{\alpha}) = (2C_T - \frac{r_H}{\alpha})\beta e^{\lambda(c\alpha K_L(1-\frac{2C_T\alpha - r_H}{r_L}))} - \frac{r_H}{\alpha}\beta e^{-\lambda(c\alpha K_L(1-\frac{2C_T\alpha - r_H}{r_L}))} < 0$.

Rearranging this inequality, we get necessary condition for $G(\frac{r_L}{\alpha}) < 0$:

$$\frac{2\alpha C_T - r_H}{r_H} < e^{-2\lambda c\alpha K_L(1-\frac{2C_T\alpha - r_H}{r_L})} \qquad \text{(S10).}$$

In summary, necessary conditions of unique positive solution are (S8), (S9) and (S10), which indicated that, in general, to have positive R_H^*, R_L^*, C_H^* and C_L^*, total consumer abundance in the system (C_T) should not be too large, which would deplete resources (R_L^* = 0); C_T also should not be too small, which would drive C_L^* to zero under fitness-dependent movement.

Appendix 2 The relationships among mobility, fitness sensitivity and the time for the system to reach equilibrium in the absence of consumer demography

In the absence of consumer demography, we used simulations to study how the time for the system to approach equilibrium depends on mobility and fitness sensitivity. Here, we define the solution approaching equilibrium when the density changes by less than 1e-6 within 10 continuous time-steps.

Without consumer movement between the two patches (i.e., $\beta = 0$), each patch would have its own equilibrium. The time to equilibrium depends on the initial densities of both consumers and resources in each patch. When consumers move ($\beta > 0$) but in random directions (i.e., no fitness sensitivity; $\lambda = 0$), the time to equilibrium depends on the density difference of consumers between the two patches. Here, we set initial densities of consumers to be equal in the two patches (i.e., no density difference of consumers), so there is no migration of consumers between the two patches when $\lambda = 0$. Therefore, in the above two scenarios (either $\beta = 0$ or $\lambda = 0$), the system has the same equilibrium and the same time to reach this equilibrium (the equilibrium time here is 53 steps; see the gray color line at $\beta = 0$ and $\lambda = 0$ in Fig. S1).

When consumers exhibit fitness-sensitive movement between the two patches (i.e., $\beta > 0$ and $\lambda > 0$), the time to equilibrium rapidly decreases as the baseline mobility increases (see the abrupt color change along β axis in Fig. S1). The time to equilibrium shows a unimodal pattern with respect to fitness sensitivity: i.e., when fitness sensitivity increases, the time to equilibrium first increases and then decreases. The unimodal pattern is stronger when the baseline mobility is relatively small (see the hump shape of time change along λ axis in Fig. S1). This unimodal pattern arises because when the fitness sensitivity becomes slightly larger than 0, the equilibrium

changes: more consumers end up in the high-quality patch than in the low-quality patch (see Fig. 1a). For this simulation, the initial densities of consumers are equal in both patches, so the system needs more time to reach the new equilibrium. Once the fitness sensitivity increases up to a certain level, consumers can move to the high-quality patch faster, thus, the time to achieve equilibrium decreases. The smaller the baseline mobility is, the stronger the influence of fitness sensitivity on the system (i.e., the hump shape along λ axis is stronger when β is smaller in Fig. S1).

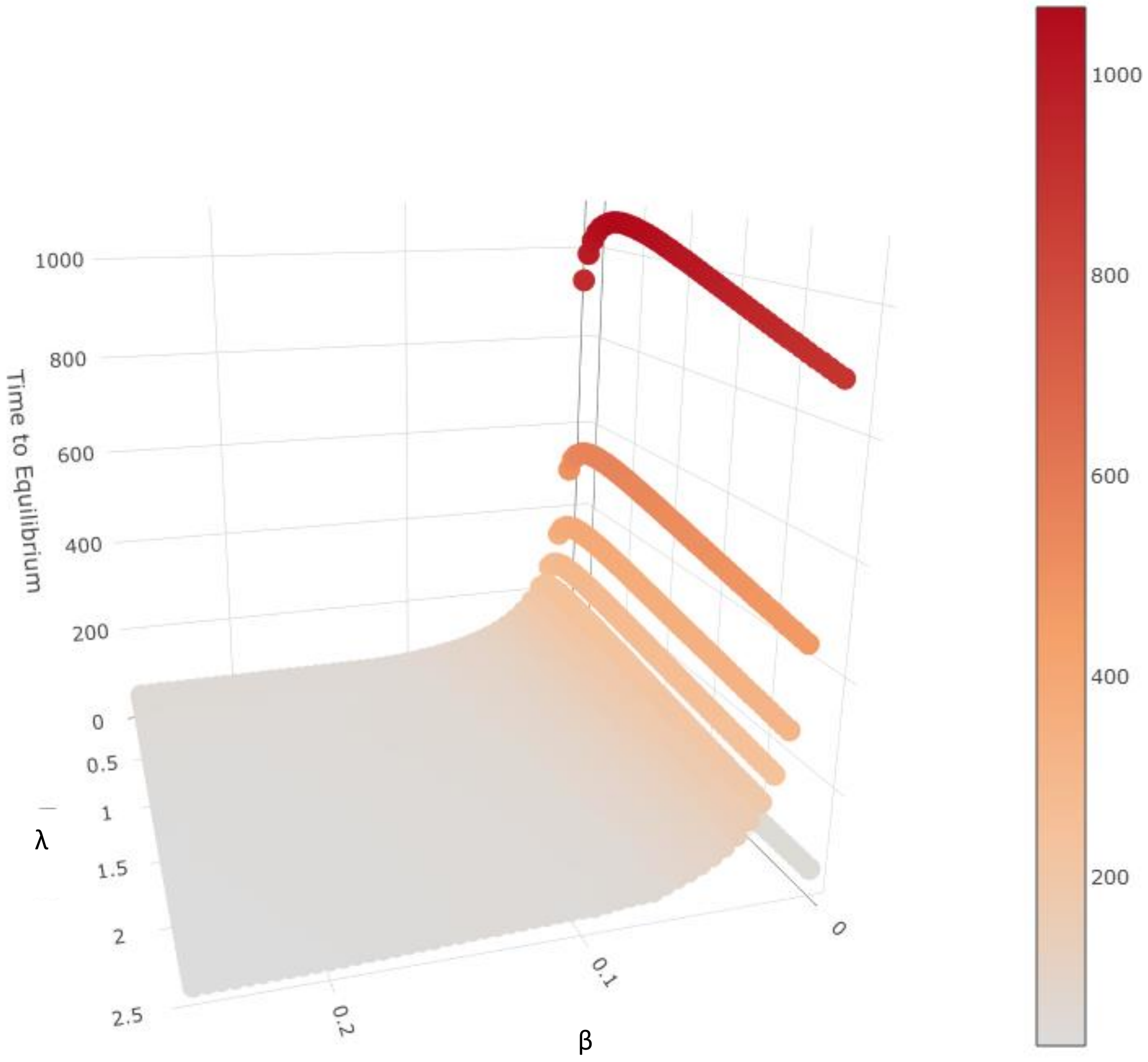

Fig. S1 The relationships among mobility (β), fitness sensitivity (λ) and the time for the system to reach equilibrium when there is no consumer demography ($p = 0$). The color bar shows the time gradient to equilibrium: from gray to dark red, time increases. The parameters are: $r_H = 2$, $r_L = 1$, $K_H = 100$, $K_L = 50$, $c = 0.05$, $\alpha = 0.05$, $\mu = 0.1$ and $C_T=15$.

Appendix 3   The proof of the relationship between the fitness sensitivity of movement and equilibria in the absence of consumer demography

Based on the model in the absence of consumer demography (S1-4), we have $R_H^* = K_H(1 - \frac{\alpha C_H^*}{r_H})$ and $R_L^* = K_L(1 - \frac{\alpha C_L^*}{r_L})$ at equilibrium, so the fitness difference of the two patches (Δ) is:

$$\Delta = R_H^* - R_L^* = \left(-\frac{\alpha K_H}{r_H} - \frac{\alpha K_L}{r_L}\right) C_H^* + K_H - K_L + \frac{\alpha K_L}{r_L} 2C_T \quad \text{(S11)}$$

Differentiating C_H^* from S11, we have $\frac{d\Delta}{dC_H^*} = -\frac{\alpha K_H}{r_H} - \frac{\alpha K_L}{r_L}$ (S12)

At equilibrium, from Eq. S3=0, we have $(2C_T - C_H^*)\, e^{\lambda c \alpha \Delta} - C_H^* e^{-\lambda c \alpha \Delta} = 0$ (S13)

Using implicit differentiation on Eq. (S13) with respect to λ and inserting Eq. (S12), we get:

$$\frac{dC_H^*}{d\lambda}\left(-e^{\lambda c \alpha \Delta} - e^{-\lambda c \alpha \Delta} - (2C_T - C_H^*)e^{\lambda c \alpha \Delta} c\alpha\lambda\left(\frac{\alpha K_H}{r_H} + \frac{\alpha K_L}{r_L}\right) - c\alpha\lambda C_H^* e^{-\lambda c \alpha \Delta}\left(\frac{\alpha K_H}{r_H} + \frac{\alpha K_L}{r_L}\right)\right) =$$
$$-(2C_T - C_H^*)e^{\lambda c \alpha \Delta} c\alpha\Delta - C_H^* e^{-\lambda c \alpha \Delta} c\alpha\Delta \quad \text{(S14)}$$

When $\Delta > 0$ (which is true for our system), from (S14), we can get:

$$\frac{dC_H^*}{d\lambda} > 0 \quad \text{(S15)}$$

From $R_H^* = K_H(1 - \frac{\alpha C_H^*}{r_H})$ and S15, we have $\frac{dR_H^*}{d\lambda} < 0$ (S16)

From S4 and S15, we have $\frac{dC_L^*}{d\lambda} < 0$ (S17)

From $R_L^* = K_L(-\frac{\alpha C_L^*}{r_L})$ and S17, we have $\frac{dR_L^*}{d\lambda} > 0$ (S18)

Inequalities S15-S18 show that with the increase of λ, more consumers would move from low-quality patch to high-quality patch ($C_H^* - C_L^*$ increases), and the disparity of resource densities would decrease ($\Delta = R_H^* - R_L^*$ decreases). This trend is always kept until $\Delta = 0$ as $\lambda \rightarrow \infty$. $\Delta = 0$ is the limiting pattern under Ideal Free Distribution (IFD).

Appendix 4 The proof of the relationships between the fitness-sensitivity of consumers' movement and regional resource density in the absence of consumer demography

From Eq. S1-S2, we get $R_H^* = K_H(1 - \frac{\alpha C_H^*}{r_H})$ and $R_L^* = K_L(1 - \frac{\alpha C_L^*}{r_L})$. By averaging these two quantities, we get the average regional density of resources, R^*:

$$R^* = \frac{R_H^* + R_L^*}{2} = \frac{1}{2}K_H\left(1 - \frac{\alpha C_H^*}{r_H}\right) + \frac{1}{2}K_L\left(1 - \frac{\alpha C_L^*}{r_L}\right)$$

$$= \frac{1}{2}\{K_H + K_L - \alpha\left(\frac{K_H}{r_H}C_H^* + \frac{K_L}{r_L}C_L^*\right)\} \quad \text{(S19)}$$

By replacing $C_L^* = 2C_T - C_H^*$, we get:

$$R^* = \frac{1}{2}\{K_H + K_L - 2\alpha\frac{K_L}{r_L}C_T - \alpha C_H^*\left(\frac{K_H}{r_H} - \frac{K_L}{r_L}\right)\} \quad \text{(S20)}$$

When $\frac{K_H}{r_H} - \frac{K_L}{r_L} = 0$, $R^* = \frac{1}{2}(K_H + K_L - 2\alpha\frac{K_L}{r_L}C_T)$, which is constant with the fixed C_T (see Fig. 2c). (S21)

When $\frac{K_H}{r_H} - \frac{K_L}{r_L} < 0$ or $r_H/K_H > r_L/K_L$, under (S15), we can get:

$$\frac{dR^*}{d\lambda} > 0 \quad \text{(S22)}$$

When $\frac{K_H}{r_H} - \frac{K_L}{r_L} > 0$ or $r_H/K_H < r_L/K_L$, under (S15), we have:

$$\frac{dR^*}{d\lambda} < 0 \quad \text{(S23)}$$
